# Echocardiographic Changes and Long-Term Clinical Outcomes in Pediatric Patients With Pulmonary Arterial Hypertension Treated With Bosentan for 72 Weeks: A *Post-hoc* Analysis From the FUTURE 3 Study

**DOI:** 10.3389/fped.2021.681538

**Published:** 2021-06-16

**Authors:** Maurice Beghetti, Rolf M. F. Berger, Damien Bonnet, Simon Grill, Catherine Lesage, Jean-Christophe Lemarie, D. Dunbar Ivy

**Affiliations:** ^1^Paediatric Cardiology Unit, Children's Hospital, Geneva, Switzerland; ^2^Centre Universitaire Romand de Cardiologie et Chirurgie Cardiaque Pédiatriques, University of Lausanne, Lausanne, Switzerland; ^3^Department of Paediatric Cardiology, Centre for Congenital Heart Diseases, Beatrix Children's Hospital, University Medical Center Groningen, University of Groningen, Groningen, Netherlands; ^4^M3C-Hospital Necker Enfants Malades, Department of Paediatric Cardiology, Université de Paris, Paris, France; ^5^Actelion Pharmaceuticals Ltd., Allschwil, Switzerland; ^6^Effi-Stat, Paris, France; ^7^Department of Pediatric Cardiology, Children's Hospital Colorado, Denver, CO, United States

**Keywords:** pulmonary arterial hypertension, pediatrics, echocardiography, vascular disease, long-term outcomes

## Abstract

FormUlation of bosenTan in pUlmonary arterial hypeRtEnsion (FUTURE) 3 was a 24-week open-label, prospective, and randomized phase 3 study that assessed the pharmacokinetics of bosentan 2 mg/kg b.i.d. or t.i.d. in children with pulmonary arterial hypertension (PAH). We report findings from a *post-hoc* analysis that explored the prognostic value of echocardiographic changes during FUTURE 3 in relation to clinical outcomes observed during the 24-week core study and 48-week extension. Patients aged ≥3 months to <12 years (*n* = 64) received oral doses of bosentan 2 mg/kg b.i.d. or t.i.d. (1:1) for 24 weeks, after which they were eligible to enter the extension with continued bosentan administration. Echocardiographic evaluations were performed at baseline, Week 12, and 24 of the core study via central reading, and analyzed *post-hoc* for correlation with clinical outcomes (time to PAH worsening, time to death, and vital status). Sixty-four patients were randomized in the core study [median (IQR) age 3.8 (1.7–7.8) years]; and 58 patients (90.6%) entered the 48-week extension. Most of the patients (68.8%) were receiving ≥1 PAH medication at baseline. Echocardiographic changes during the core study were small but with high variability. There were statistically significant associations at Week 24 between worsening of the parameters, systolic left ventricular eccentricity index (LVEIS) and E/A ratio mitral valve flow, and the outcomes of time to death and time to PAH worsening. Additional studies that utilize simple and reproducible echocardiographic assessments are needed to confirm these findings and subsequently identify potential treatment goals in pediatric PAH.

## Introduction

In children, pulmonary arterial hypertension (PAH) is a rare but progressive disease that ultimately leads to right ventricular failure and premature death (1–5). Echocardiography is integral to the screening and clinical management of patients with PAH, as it captures the anatomy and function of both the right and left sides of the heart during systole and diastole (3–6). However, there is no consensus on which variables to use in pediatric PAH. Anatomical and functional echocardiographic measures of both the right and left sides of the heart have been reported to correlate consistently with disease severity and clinical outcome measures including WHO functional class (FC), hemodynamic measures, and survival in pediatric PAH (7–11). Although treatment-induced changes in echocardiographic parameters [tricuspid annular plane systolic excursion (TAPSE)] have been shown to be associated with improved survival (12), the value of changes in echocardiographic parameters to predict clinical outcomes in pediatric patients has not been fully defined to date (4, 5, 9, 11).

Bosentan (Tracleer®), an oral dual endothelin receptor antagonist, is one of the first therapies to be approved in the United States and European Union for the treatment of pediatric PAH. The FormUlation of bosenTan in pUlmonary arterial hypeRtEnsion (FUTURE) 3 study investigated whether bosentan exposure might be increased to levels observed in adults when administered t.i.d. rather than b.i.d. (13). Echocardiographic assessments were conducted at screening and Weeks 12 and 24 of the core study, and several exploratory clinical outcomes were assessed over the course of the 24-week core study and subsequent 48-week extension (13). These data may allow identification of potential treatment goals in pediatric PAH, as opposed to assessment of prognostic value based on echocardiographic measurements at a single time point.

This *post-hoc* analysis explored possible correlations between echocardiographic changes during the 24-week FUTURE 3 core study and clinical outcomes observed across the core plus 48-week extension treatment period, totaling 72 weeks.

## Materials and Methods

### Patients and Future 3 Study Design

This *post-hoc* analysis was based on data from patients in FUTURE 3, a multinational, open-label, and prospective phase 3 study conducted in 30 centers (NCT01223352). The full eligibility criteria and methodology of FUTURE 3 have been published (13). Briefly, pediatric patients aged ≥3 months to <12 years who had PAH confirmed by right-sided heart catheterization were randomized 1:1 to receive the oral pediatric formulation of bosentan at a dose of 2 mg/kg administered either b.i.d. (4 mg/kg total daily dose) or t.i.d. (6 mg/kg total daily dose) for 24 weeks. All the patients were included in the *post-hoc* analysis. The patients who completed a 24-week end-of-study visit while still on bosentan became eligible to participate in the 48-week follow-up extension (total treatment duration of up to 72 weeks). Those who did not enter the extension underwent continued safety monitoring during a 60-day post-treatment follow-up period after the 24-week study visit.

The protocols of the FUTURE 3 study (plus the extension) were reviewed and approved by the ethics committees or institutional review boards shown in Supplementary Table 1. The study was conducted in accordance with the Declaration of Helsinki and the clinical trial regulations of each country. Written informed consent was obtained from all the parents or legal representatives.

### Parameters and Outcomes

During the core study, echocardiography/Doppler measurements were performed in all the patients at screening, week 12 and 24, or end-of-core-study visit premature discontinuation. Anatomical and functional echocardiographic parameters for the right side of the heart included right ventricular fractional area change (RVFAC) based on right ventricular end-diastolic and end-systolic areas; right ventricular systolic pressure (RVSP); TAPSE normalized for body surface area (BSA); and inferior vena cava size collapse (IVCC) based on the change in size between inspiration and expiration. Parameters for the left side of the heart were also analyzed: systolic and diastolic left ventricular eccentricity indices (LVEIS and LVEID, respectively), and E/A ratio mitral valve flow. Evaluations of all echocardiographic parameters were standardized via central reading and independent adjudication by echocardiography experts that resulted in either “silent approval” or discussion between the central reader and adjudicators in case of disagreement.

Three main clinical outcomes were evaluated during both the 24-week core study and the 48-week extension as follows: on-treatment, vital status (alive or dead) was assessed up to the completion of the 48-week extension (EOS). Time to death was evaluated using an intention-to-treat approach, with patients last known to be alive censored based on the date of last contact up to EOS; time to PAH worsening (defined as lung transplant, hospitalization for PAH progression, initiation of a new PAH therapy, new/worsening right-sided heart failure or death) was analyzed up to the end of treatment plus 7 days (EOT + 7 days) or death; and the patients without a PAH worsening event were censored at EOT + 7 days.

### Statistical Analyses

All statistical evaluations in the *post-hoc* analysis were exploratory. For each echocardiographic parameter, “worsening” was defined as the percentage increase or decrease from baseline as follows: increase at Week x = [(Week x/baseline) – 1]^*^100 for RVSP, LVEID, and LVEIS; decrease at Week x = [1 – (Week x/baseline)]^*^ 100 for TAPSE, IVCC, RVFAC, and E/A ratio mitral valve flow. The geometric mean of “worsening” was derived as [geometric mean of (Week x/baseline) – 1]^*^ 100 for RVSP, LVEID, and LVEIS, and as [1 – geometric mean of (Week x/baseline)]^*^ 100 for TAPSE, IVCC, RVFAC, and E/A ratio mitral valve flow. All analyses were based on the all-randomized and echo/Doppler sets, including the all-randomized patients except those with PAH-CHD featuring systemic-to-pulmonary shunts, or Eisenmenger syndrome, whether or not they received study medication.

Associations between worsening of echocardiographic parameters from baseline at Week 12 or 24 and vital status at EOS were investigated using logistic regression and odds ratios (OR) with 90% two-sided confidence limits. Associations between worsening from baseline at Week 12 or 24, and both time to death and time to first PAH worsening were investigated using a Cox model and summarized using the corresponding hazard ratio (HR) and 90% two-sided confidence limits. Both for logistic regressions and Cox models, “worsening” was expressed on a base 2 logarithmic scale as Log2 (Week x/baseline) for RVSP, LVEID, and LVEIS, and as –Log2 (Week x/baseline) for TAPSE, IVCC, RVFAC, and E/A ratio mitral valve flow. This way, the OR and the HR represent, respectively, the odds increase and the risk increase associated with doubling in the echocardiography parameter for RVSP, LVEID, and LVEIS, and to halving in the echocardiography parameter for TAPSE, IVCC, RVFAC, and E/A ratio mitral valve flow. Due to the low number of events, both OR and HR estimations were performed by penalized maximum likelihood estimation for all logistic regressions and Cox models based on published methods (14, 15). For these exploratory analyses, the significance level was set to 10% (*p* < 0.1).

Additionally, a categorical analysis of the associations between percent worsening from baseline in echocardiographic parameters and clinical outcomes was conducted, where two different threshold changes (10 and 20%) in echocardiographic values were considered. For each threshold, worsening at Week x was used as an explanatory variable and was defined in binary form (i.e., worsening < threshold and worsening ≥ threshold).

Association analyses were performed with regard to observed cases only, with no imputation applied. As there were no differences observed in the age groups or exposure of the b.i.d. and t.i.d. treatment groups during the FUTURE 3 study (13), this *post-hoc* analysis did not distinguish between treatment arms or patient age categories. The patients were sub-grouped according to the presence or absence of CHD associated with open systemic-to-pulmonary shunts, and into treatment-naïve or non-treatment-naïve patients.

## Results

### Patients and Treatment

Sixty-four patients were randomized (all randomized set) to bosentan b.i.d. or t.i.d. in the 24-week core study (Supplementary Figure 1); eight patients had CHD associated with systemic-to-pulmonary shunts, therefore the echo/Doppler set included 56 patients (87.5%). Fifty-eight patients (90.6%) entered the 48-week extension and 45 patients (70.3%) completed the extension phase of the study. In this study, 13 patients (20.3%) discontinued the bosentan treatment prematurely during the extension, with 11 discontinuing because of adverse events.

Demographic and clinical baseline characteristics of the all-randomized set are summarized in Table 1. The median (interquartile range (IQR)) age was 3.8 (1.7–7.8) years, and the patients were predominantly male and Caucasian. The most frequent PAH etiologies were idiopathic PAH (IPAH) (46%) or associated PAH (APAH) (38.1%). Eighteen (28.1%) had previously received bosentan with or without other drugs. The median (IQR) bosentan exposure was 72.4 (61.6–75.2) weeks.

**Table 1:** Patient characteristics at baseline in the FUTURE 3 trial (all randomized patients; *N* = 64).

**Characteristic**	
**Sex, n (%)**	
Male	36 (56.3)
Female	28 (43.8)
**Age, years**	
Mean (SD)	4.8 (3.6)
Median (range)	3.8 (0.3–11.4)
Median (IQR)	3.8 (1.7–7.8)
**Race, n (%)**	
Caucasian/white	48 (75.0)
Black	3 (4.7)
Asian	10 (15.6)
Hispanic	1 (1.6)
Other	2 (3.1)
**BMI, kg/m**^**2**^	
Mean (SD)	15.0 (2.5)
Etiology^*^, n (%)	
IPAH	29 (46.0)
HPAH	2 (3.2)
APAH	24 (38.1)
PAH-CHD^†^	8 (12.7)
**WHO functional class, n (%)**	
I	19 (29.7)
II	27 (42.2)
III	18 (28.1)
**Global Clinical Impression Scale^‡^, n (%)**	
Very bad	3 (4.7)
Bad	8 (12.5)
Neither good nor bad	11 (17.2)
Good	30 (46.9)
Very good	12 (18.8)
**PAH-specific medications, n (%)**	
PDE-5 inhibitor	23 (35.9)
Bosentan	7 (10.9)
Bosentan + prostanoid + PDE-5 inhibitor	7 (10.9)
Bosentan + PDE-5 inhibitor	4 (6.3)
Prostanoid	1 (1.6)
None	22 (34.4)

* *data are missing for n = 1*;

† *open shunt;physician-rated*;

‡ *values are numbers (n) and percentages of patients unless otherwise stated. APAH, associated PAH; BMI, body mass index; HPAH, heritable PAH; IPAH, idiopathic PAH; IQR, interquartile range; PAH, pulmonary arterial hypertension; PDE-5, phosphodiesterase-5; SD, standard deviation; WHO, World Health Organization*.

In total, 42.2% of the patients were in WHO FC II at baseline, while approximately equal proportions (close to 30% each) were in WHO FCs I and III. At Week 72 in the all-randomized set, WHO FC was stable for 50 patients (78.1%), had improved for six patients (9.4%), and worsened for eight patients (12.5%), and in the echo/Doppler set was stable for 46 patients (86.8%), had improved for seven patients (13.2%), and worsened for two patients (3.6%). Fifteen patients (23.4%) in the all-randomized set experienced worsening of PAH by EOT + 7; and this included 11 patients (17.2%) with new/worsening right-sided heart failure, 10 patients (15.6%) who died, 7 patients (10.9%) who were hospitalized because of PAH progression, and 4 patients (6.3%) who initiated new therapy for PAH. In the echo/Doppler set, 12 patients (21.4%) experienced PAH worsening. Twelve patients (18.8%) in the all-randomized set and 11 patients (19.6%) in the echo/Doppler set had died by the EOS.

### Absolute Changes From Baseline in Echocardiographic Parameters

Absolute changes from baseline in echocardiographic parameters in the all-randomized set (*N* = 64) and the subgroup excluding patients with open shunts (echo/Doppler set; *N* = 56) are listed in Table 2. Although variable, there were small but consistent improvements at Week 24 (3.7–24.8%) in the IVCC, E/A ratio mitral valve flow, LVEID, and LVEIS of both groups.

**Table 2:** Changes in echocardiographic parameters from baseline in the all-randomized set and excluding patients with open systemic-to-pulmonary shunts.

**Parameter**	**Time**	All-randomized set^*^ (*N* = 64)			**Excluding patients with open shunts (*N* = 56)**		
		**n**	**Mean (SD) baseline^†^**	**Mean change from baseline (95% CI)**	**n**	**Mean (SD) baseline^†^**	**Mean change from baseline (95% CI)**
RVFAC	W12	27	33.980 (12.717)	−2.183 (−8.563, 4.197)	23	33.389 (13.523)	−1.781 (−9.300, 5.737)
	W24	22	36.004 (13.260)	−3.106 (−9.196, 2.983)	19	35.671 (14.055)	−2.395 (−9.451, 4.662)
IVCC	W12	43	49.308 (19.136)	10.047 (5.208, 14.886)	37	47.152 (18.897)	10.841 (5.739, 15.944)
	W24	33	50.139 (20.944)	7.368 (−0.295, 15.031)	28	46.855 (20.910)	8.969 (0.018, 17.919)
LVEID	W12	45	1.533 (0.451)	−0.063 (−0.190, 0.063)	40	1.540 (0.468)	−0.081 (−0.221, 0.059)
	W24	26	1.504 (0.453)	−0.050 (−0.228, 0.129)	22	1.509 (0.467)	−0.070 (−0.279, 0.139)
LVEIS	W12	46	1.866 (0.946)	−0.199 (−0.408, 0.009)	40	1.839 (0.900)	−0.157 (−0.368, 0.055)
	W24	27	1.863 (0.956)	−0.109 (−0.538, 0.320)	22	1.799 (0.845)	−0.026 (−0.519, 0.467)
E/A ratio mitral valve flow	W12	34	1.378 (0.442)	0.071 (−0.060, 0.201)	30	1.410 (0.460)	0.051 (−0.091, 0.193)
	W24	25	1.443 (0.457)	0.210 (−0.079, 0.500)	21	1.513 (0.461)	0.252 (−0.092, 0.597)
RVSP	W12	44	69.540 (24.747)	0.890 (−6.075, 7.854)	36	69.124 (24.940)	−0.040 (−7.144, 7.065)
	W24	32	67.933 (26.891)	0.007 (−9.301, 9.315)	25	67.669 (27.570)	−1.094 (−12.042, 9.854)
TAPSE	W12	43	2.615 (1.013)	−0.077 (−0.236, 0.082)	35	2.552 (1.090)	−0.087 (−0.246, 0.071)
	W24	28	2.601 (0.856)	−0.196 (−0.490, 0.097)	22	2.513 (0.905)	−0.276 (−0.630, 0.078)

*Data are without imputations*.

* *including patients with systemic-to-pulmonary shunts*;

† *mean (SD) baseline values for the number of patients (indicated in the n = cell of each row) who also had data available for that parameter at Week 12 or 24 of the core study*.

*BSA, body surface area; IVCC, inferior vena cava size collapse; LVEID, diastolic left ventricular eccentricity index; LVEIS, systolic left ventricular eccentricity index; RVFAC, right ventricular fractional area change; RVSP, right ventricular systolic pressure; TAPSE, tricuspid annular plane systolic excursion*.

### Associations Between Worsening From Baseline in Echocardiographic Parameters and Clinical Outcomes

**Figure 1:**
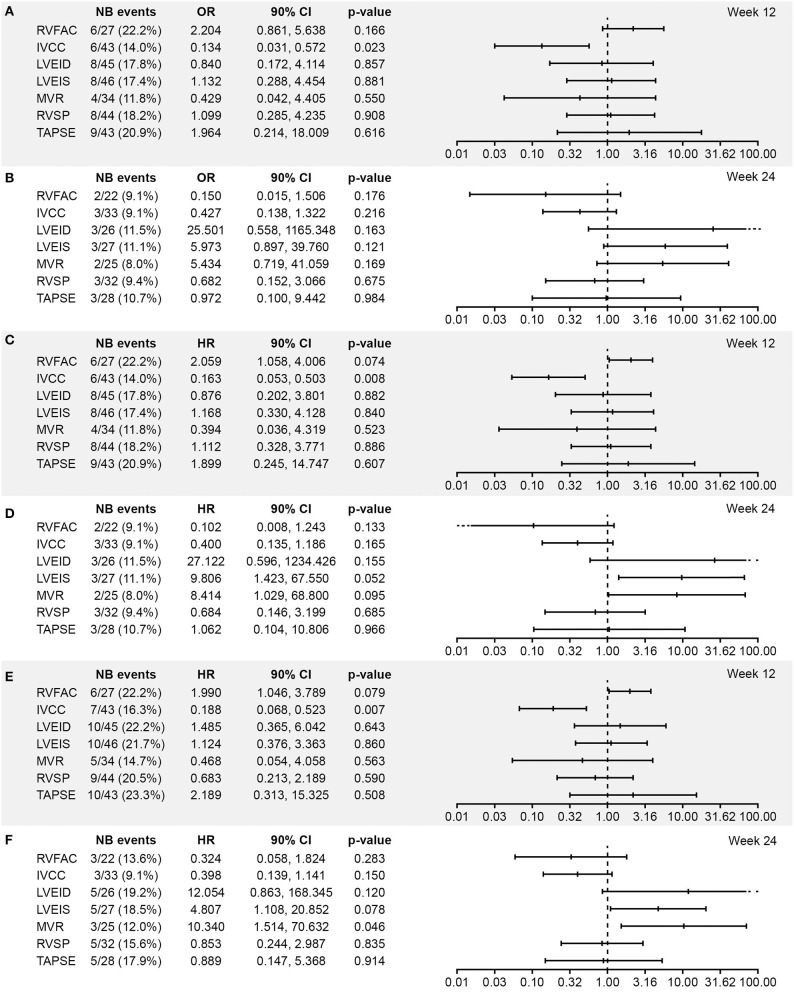
Association between worsening from baseline in echocardiographic parameters and **(A,B)** vital status at EOS, **(C,D)** time to death, and **(E,F)** time to PAH worsening at Weeks 12 and 24, respectively (all randomized set; *N* = 64). Analysis based on logistic regression for ORs and on Cox models for HRs. The x-axis shows the estimate of the OR or HR (center tick mark) with its 90% confidence limits (left and right tick marks). The estimate quantifies the increase in odds (for OR) or in risk (for HR) of experiencing the clinical end point in case of a worsening of the echocardiography parameter corresponding to a doubling of the measure (for LVEID, LVEIS and RVSP (Log2[Wk x/Bsl])) or to a halving of the measure (for RVFAC, IVCC, MVR and TAPSE (-Log2[Wk x/Bsl])). An OR or HR with a *p* < 0.1 indicates a statistically significant correlation between the echocardiographic parameter and the considered end point (vital status at EOS, time to death up to EOS, and time to first PAH worsening up to EOT +7 days). BSA, body surface area; EOS, end of study; EOT, end of treatment; RVFAC, right ventricular fractional area change; HR, hazard ratio; IVCC, inferior vena cava size collapse; LVEID, diastolic left ventricular eccentricity index; LVEIS, systolic left ventricular eccentricity index; MVR, E/A ratio mitral valve flow; OR, odds ratio; RVSP, right ventricular systolic pressure; TAPSE, tricuspid annular plane systolic excursion.

Associations between worsening from baseline in echocardiographic parameters and clinical outcomes in the all-randomized set are presented as forest plots in Figure 1. Echocardiographic parameters that were statistically significantly (OR/HR > 1 and *p* < 0.1) associated with clinical outcomes were worsening of RVFAC at Week 12, worsening of LVEIS at Week 24, and worsening of E/A ratio mitral valve flow at Week 24, all of which were associated with outcomes time to death and time to PAH worsening (Figure 1). A significant association was also observed between worsening of IVCC at Week 12 and all the three clinical outcomes (OR/HR < 1 and *p* < 0.1). There were also trends toward associations between worsening of LVEID at Week 24 and vital status, time to death, and time to PAH worsening in the all-randomized set, but the ORs had extremely wide 90% CIs (Figure 1).

**Figure 2:**
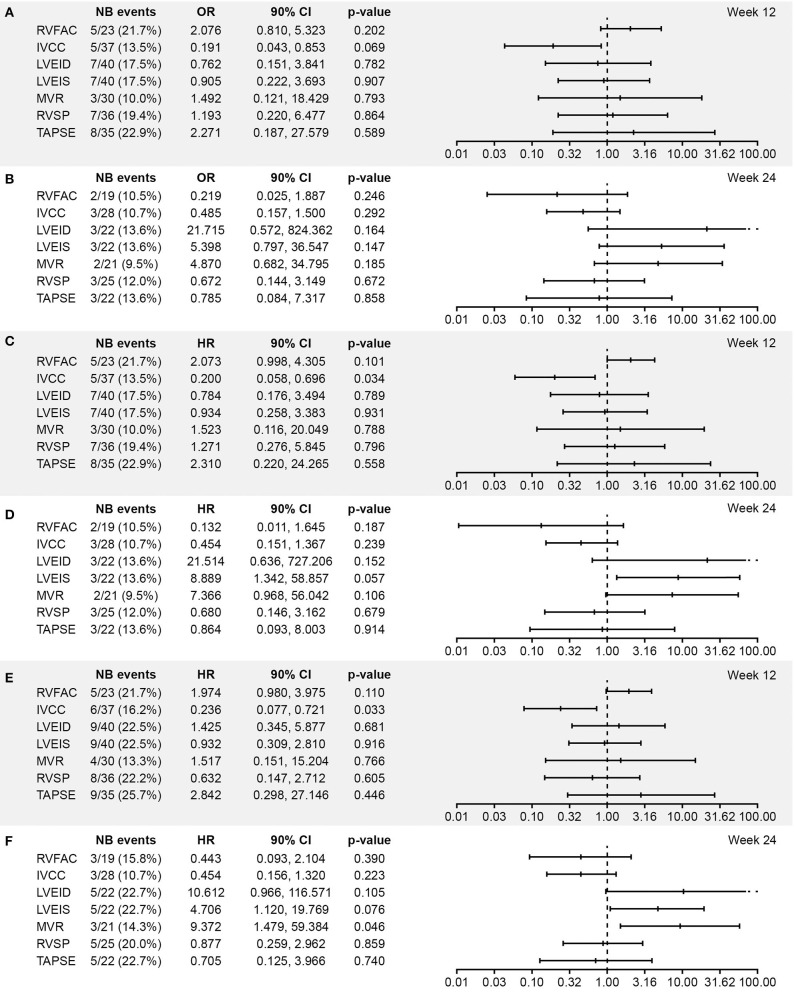
Association between worsening from baseline in echocardiographic parameters and **(A,B)** vital status at EOS, **(C,D)** time to death, and **(E,F)** time to PAH worsening at Week 12 and 24, respectively (excluding patients with shunts, i.e., the echo/Doppler set; *N* = 56). Analysis based on logistic regression for ORs and on Cox models for HRs. The x-axis shows the estimate of the OR or HR (center tick mark) with its 90% confidence limits (left and right tick marks). The estimate quantifies the increase in odds (for OR) or in risk (for HR) of experiencing the clinical end point in case of worsening of the echocardiography parameter corresponding to doubling of the measure [for LVEID, LVEIS, and RVSP (Log2[Wk x/Bsl]) or to halving of the measure [for RVFAC, IVCC, MVR, and TAPSE(–Log2[Wk x/Bsl]). An OR or HR with a *p* < 0.1 indicates a statistically significant correlation between the echocardiographic parameter and the considered end point (vital status at EOS, time to death up to EOS, and time to first PAH worsening up to EOT + 7 days). BSA, body surface area; EOS, end of study; EOT, end of treatment; RVFAC, right ventricular fractional area change; HR, hazard ratio; IVCC, inferior vena cava size collapse; LVEID, diastolic left ventricular eccentricity index; LVEIS, systolic left ventricular eccentricity index; MVR, E/A ratio mitral valve flow; OR, odds ratio; RVSP, right ventricular systolic pressure; TAPSE, tricuspid annular plane systolic excursion.

ORs and HRs for worsening in echocardiographic parameters in relation to each of the three clinical outcomes in the echo/Doppler set were similar to those in the all-randomized set (Figure 2).

The categorical statistical analysis (using thresholds of 10 and 20% for worsening from baseline) identified significant associations between worsening of LVEID (20% threshold) at Week 24 and vital status and time to death (Supplementary Figures 2–4). A significant association between LVEIS at Week 24 (at both 10 and 20% thresholds) and vital status was also observed, along with a trend toward an association between LVEIS at Week 24 (at both 10 and 20% thresholds) and time to death (Supplementary Figures 2–4). Worsening of E/A ratio mitral valve flow (20% threshold) at Week 24 was significantly associated with time to PAH worsening (Supplementary Figures 2–4).

### Absolute Changes From Baseline in Echocardiographic Parameters in Treatment-Naïve vs. Non-naïve Patients

In total, 42 patients (65.6%) had received previous treatment with bosentan, prostanoids, phosphodiesterase-5 (PDE-5) inhibitors, or combinations thereof, before entering the FUTURE 3 core study, and 22 patients (34.4%) were naïve to PAH treatment. In the non-naïve subgroup [median (IQR) age 4.9 (1.7–8.3) years], 19 patients (46.3%) had IPAH, 17 (41.5%) had APAH, and three (7.3%) had PAH-CHD with open shunts, and the proportions of patients in WHO FC categories I, II, and III were 19, 54.8, and 26.2%, respectively. In the treatment-naïve subgroup [median (IQR) age 3.4 (1.8–4.8) years], 10 patients (45.5%) had IPAH, 7 (31.8%) had APAH, and 5 (22.7%) had PAH-CHD with open shunts, and the proportions of patients in WHO FC categories I, II, and III were 50, 18.2, and 31.8%, respectively.

Data from echocardiographic assessments in the non-naïve subgroup vs. the naïve subgroup are summarized in Supplementary Table 2. Changes in absolute mean echocardiographic parameters in these subgroups were generally similar to those in the all-randomized set. Only IVCC showed a marked difference between these subgroups by Week 24 of treatment: 33% improvement in non-naïve patients vs. 3.1% improvement in treatment-naïve patients.

## Discussion

In the *post-hoc* analysis of pediatric patients with PAH from the FUTURE 3 study, echocardiographic changes in over 24 weeks of treatment with bosentan b.i.d. or t.i.d. were small and featured a high degree of variability. However, we identified some statistically significant associations between worsening from baseline in echocardiographic parameters measured over 24 weeks and the selected clinical outcomes measured after 72 weeks in total.

Based on findings from the analysis of worsening from baseline in echocardiographic parameters, an increase in LVEIS and E/A ratio mitral valve flow, and to a lesser extent, LVEID, may be associated with an increased risk of death or PAH worsening in pediatric patients with PAH. To a degree, these findings corroborate previously published data regarding diastolic and systolic LV indices as valid echocardiographic measures for assessing RV volume and/or pressure overload in both adults and children (9, 16, 17). The right ventricular/left ventricular ratio may also be relevant (8, 9, 18).

Given the lack of consensus on which echocardiographic variables to use in pediatric PAH, it is important to highlight that the findings on eccentricity index are consistent with the findings of others, such as those from a recent prospective study of LV shape measures in 78 pediatric patients with PH (70.5% PAH) by concurrent echocardiography and cardiac catheterization (19). In that study, Burkett et al. demonstrated that eccentricity index and right-to-left ventricular ratio correlated with invasive simultaneous hemodynamic measurements, PH-related hospitalizations, BNP/NT-proBNP levels, and WHO FC (19). LV dysfunction is of increasing interest in pediatric PAH, since its prognostic relevance has become more apparent (20), and it may reflect LV compression as a result of RV enlargement (21). Burkett et al. demonstrated the strongest correlations for maximal eccentricity index, a new and highly reproducible measure (19). Data also show that the use of LVEID and LVEIS may reduce inter-observer variability in PAH (7). Additional echocardiographic parameters have been proposed since FUTURE 3 was conducted, speckle-tracking strain analysis, for example (22). However, none have been identified to have overcome the inter-observer/center variability observed in this study. The findings add to the evidence that the eccentricity index may have a prognostic value in pediatric PAH, and further evaluation is warranted, particularly given the reproducibility of this parameter.

Several other (pediatric and adult) studies have indicated that echocardiographic parameters for the left side of the heart offer prognostic information on PAH (9, 23, 24), although most of the previous studies have focused on anatomic and functional variables for the right side of the heart (8, 10, 17, 25–28). In a systematic review and meta-analysis of prognostic factors in pediatric PAH, Ploegstra et al. identified nine echocardiographic parameters where a single measurement was associated with length of survival in children, all of which were related to the function of the right side of the heart (26). In addition, treatment-induced changes in TAPSE have been shown to be associated with improved survival in treatment-naïve pediatric patients with PAH who started taking PAH-targeted drugs (12). While the relevance of the parameters for the right side of the heart in PAH screening, diagnosis, and monitoring is well established, there are relatively few data on the parameters for the right side of the heart that have been studied in the same way in more than one study, which prevents an adequate comparison. Ideally, a longitudinal study should be conducted, wherein new analyses could be performed on the same group of patients.

The unmet need for a suitable non-invasive primary end point for pediatric PAH trials has been the driving force for much of the research on the prognostic value of echocardiographic parameters for this rare disease, but conducting such studies is difficult (1). Changes in echocardiographic parameters have never been used as a primary end point in adult PH trials, and a 2019 report from a multi-stakeholder meeting on pediatric PAH involving FDA and EMA regulators highlighted the inter-observer variability with echocardiographic assessment and confirmed this was not considered a feasible primary end point (29). However, longitudinal echocardiographic data are a key to guiding the long-term management of PAH. Therefore, it is very important to identify and combine reproducible parameters to establish a key set of prognostic indicators for use in clinical practice and clinical studies as non-primary end points. The evidence base is growing, but current recommendations are largely based on few single-center non-randomized studies with different designs (22). While the strongest level of recommendation is given for TAPSE, a well-established indicator of systolic longitudinal RV function in pediatric PAH, overreliance on a single measure undermines the validity of the assessment (22). TAPSE also does not measure apical RV function (22). Similarly, a 2016 update from the American Society of Echocardiography and the European Association of Cardiovascular Imaging recommends using several parameters to evaluate LV diastolic function (30). Worsening of E/A ratio mitral valve flow (described herein) could suggest a change in diastolic LV function, but further evaluation would be required to confirm this. Future studies should take a multi-parametric approach that includes reproducible echocardiographic measures to define the optimal combination to measure.

Challenges related to echocardiographic assessments were addressed in this study using several strategies. This analysis assessed the entire study population using seven widely used echocardiographic parameters that have been shown to be of clinical value in PAH screening and diagnosis, and/or for prognosis in pediatric PAH, although it should be noted that much more data on prognostic echocardiographic parameters have been published since FUTURE 3 was designed (22, 31), and that there is no consensus on which parameters to use. Inter-observer variability was minimized by rigorous training across study sites, central assessment of echocardiographic findings with independent adjudication, and conducting a subgroup analysis that excluded patients with systemic-to-pulmonary shunts. Ideally, future studies should be longitudinal in design, and they should implement these protocols for the echocardiographic assessment of pediatric PAH, wherever possible, particularly the central reading of echocardiograms. Given that body size is a powerful determinant of cardiovascular dimensions (32), measurements relative to BSA were used where appropriate (e.g., for tricuspid valve movement during systole) (33, 34). Nevertheless, the difficulty of performing echocardiograms according to standardized protocols resulted in variable data.

The small magnitude of the changes in clinical outcomes and echocardiographic parameters observed in the FUTURE 3 participants means a large number of patients are required to observe a difference, but the rarity of pediatric PAH precludes study populations from being much larger than that described here. These small effect sizes likely hampered the ability to identify associations. This is important to take into account when considering that the study did not identify significant associations between change in TAPSE and outcome measures. The FUTURE 3 study population was also heterogeneous in terms of varied medical histories, prior treatment regimens, disease etiology, and WHO FC. Furthermore, this analysis was not pre-specified; it was *post-hoc* in nature. The FUTURE 3 study was not designed or powered to demonstrate changes in echocardiographic variables or associations with clinical outcome. While challenging, echocardiographic sub-studies/investigations are feasible and very valuable, although echocardiographic parameters are not viable as a primary end point for pediatric PAH trials (29).

This exploratory analysis has several limitations, such as lack of a placebo arm, which prevented full insight into statistical associations. The exact dosing of bosentan varied slightly around the target dose of 2 mg/kg^−1^, since the smallest dose unit was 8 mg (a quarter of a tablet), possibly contributing to variability. No data imputation was applied for missing values, which helped to avoid bias, but it also reduced a small data pool and may have increased the possible influence of variability.

## Conclusion

This *post-hoc* analysis identified a statistically significant association between worsening in systolic left ventricular eccentricity index and E/A ratio mitral valve flow at Week 24 and increased risk of death or disease worsening in pediatric patients with PAH. We recognize that the FUTURE 3 study was not powered to demonstrate changes in echocardiographic parameters. Despite echocardiographic evaluations being standardized via central reading and independent adjudication, and the size and length of this pediatric PAH trial being relatively large for the field, the analysis was hampered by high variability in echocardiographic data as well as small changes from baseline in echocardiographic measurements and clinical outcomes (reflecting the fact that most of the patients remained clinically stable during the study). Further studies are required to elucidate potential treatment goals in pediatric PAH.

## Data Availability Statement

The data sharing policy of Janssen Pharmaceutical Companies of Johnson & Johnson is available at https://www.janssen.com/clinicaltrials/transparency. As noted on this site, requests for access to the study data can be submitted through Yale Open Data Access (YODA) Project site at http://yoda.yale.edu.

## Ethics Statement

The studies involving human participants were reviewed and approved by relevant ethics committees or institutional review boards at each of the participating institutions (Supplementary Table 1). The study was conducted in accordance with the Declaration of Helsinki and clinical trial regulations of each country. Written informed consent to participate in this study was provided by the participants' legal guardian/next of kin.

## Author Contributions

MB, RB, DB, SG, CL, and DI contributed to the conception and design of the study and interpretation of data. J-CL performed statistical analysis. All the authors contributed to manuscript revision and read and approved the submitted version.

## Conflict of Interest

SG and CL are employed by Actelion Pharmaceuticals Ltd., a Janssen Pharmaceutical Company of Johnson & Johnson, and declare that this study received funding from Actelion Pharmaceuticals Ltd., a Janssen Pharmaceutical Company of Johnson & Johnson. The sponsor was involved in the conception and design of the study, analysis and interpretation of data, critical revision of the manuscript, and approval for submission to the journal. J-CL is employed by Effi-Stat, a contract research organization that received financial support from Janssen Pharmaceutical Companies of Johnson & Johnson for statistical analysis and programming in this study. The disclosures of the remaining authors are as follows: MB has received grants, personal fees, and non-financial support from Janssen Pharmaceutical Companies of Johnson & Johnson, and has received personal fees from Bayer HealthCare Pharmaceuticals LLC, Bristol–Myers Squibb, Eli Lilly and Company, and GlaxoSmithKline. RB's institution (University Medical Center Groningen) contracts with Janssen Pharmaceutical Companies of Johnson & Johnson, and Eli Lilly and Company for RB to be a consultant, and has received research support from Janssen Pharmaceutical Companies of Johnson & Johnson. DB has received personal fees from Janssen Pharmaceutical Companies of Johnson & Johnson, Bayer HealthCare Pharmaceuticals LLC, Bristol–Myers Squibb, Eli Lilly and Company, and Novartis. DI's institution (The University of Colorado) contracts with Janssen Pharmaceutical Companies of Johnson & Johnson, Bayer HealthCare Pharmaceuticals LLC, GlaxoSmithKline, Lilly, and United Therapeutics for DI to be a consultant.
