# Supplementary material for: Echocardiographic Changes and Long-Term Clinical Outcomes in Pediatric Patients With Pulmonary Arterial Hypertension Treated With Bosentan for 72 Weeks: A Post-hoc Analysis From the FUTURE 3 Study
Source: Front Pediatr. 2021 Jun 16;9:681538. doi: 10.3389/fped.2021.681538 (PMC8242164; doi:10.3389/fped.2021.681538)

**Echocardiographic changes and long-term clinical outcomes in pediatric patients with pulmonary arterial hypertension treated with bosentan for 72 weeks: a *post hoc* analysis from the FUTURE 3 study**

**Supplementary material**

**Supplementary Table S1:** List of Independent Ethics Committees and Institutional Review Boards

| **Centre** | **Address** | **Approval Number** |
| --- | --- | --- |
| **Germany** |  |  |
| 1403 (Central) | Landesamt für Gesundheit und Soziales Geschäftsstelle der Ethik-Kommission des Landes Berlin | 10/0432-ZSEK13 |
| 1404 (Central) | Landesamt für Gesundheit und Soziales Geschäftsstelle der Ethik-Kommission des Landes Berlin |  |
| **Italy** |  |  |
| 1502 (Local) | Comitato Etico per la Sperimentazione Clinica dell’IRCCS Ospedale Pediatrico Bambino Gesù di Roma | CE353 |
| **Spain** |  |  |
| 1906 (Local) | Hospital Universitario La Paz – Ethics Committee Paseo de la Castellana 261 | RD223/2004 |
| 1907 (Central) | Hospital Vall d’ Hebrón, Fundacio per la Recerca | OFSAN10000 |
| **France** |  |  |
| 2201 (Central) | CPP Ile-de-France II  Laboratoire d'éthique médicale et médecine légale | 2010-09-01 |
| 2202 (Central) | CPP Ile-de-France II  Laboratoire d’éthique médicale et médecine légale | A101289-16 |
| **Belarus** |  |  |
| 3001 (Local) | LEC of the Republican Scientific-Practical Center “Cardiology” | Primary submission (Prot V1): No.1/01.11  Protocol V2: Decision #19/05.06.12 |
| **Czech Republic** |  |  |
| 3301 (Central) | Multicentrická Etická komise FN Motol | C02KX01 |
| **Hungary** |  |  |
| 3401 (Central) | Egészségügyi Tudományos Tanács Klinikai Farmakológiai Etikai Bizottság (Medical Research Council Ethics Committee for Clinical Pharmacology) | 201013/SA1 |
| 3402 (Central) | Egészségügyi Tudományos Tanács Klinikai Farmakológiai Etikai Bizottság (Medical Research Council Ethics Committee for Clinical Pharmacology) |  |
| **Poland** |  |  |
| 3604 (Central) | Komisja Bioetyczna przy Instytucie “Pomnik Centrum Zdrowia Dziecka” | 135/KBE/2011 |
| 3605 (Central) | Komisja Bioetyczna przy Instytucie “Pomnik Centrum Zdrowia Dziecka” |  |
| **Russian Federation (CEC)** |  |  |
| (Central) | Ethics Council of the Ministry of Health of Russia  Central IRB/IEC, Rahmanovsky pereulok, GSP-4, 127994 Moscow, Russian Federation | Protocol 1: Extract from Protocol 12 dated 02.03.2011 (internal No 40850)  Prot 2: 27.06.2012 / Extract from EC meeting minutes №42 |
| 3801 (Local) | State Budgetary Educational Institution of Higher Professional Education | 3801: Protocol 1: 16.05.2011/  LEC meeting minutes (m.m.) #4, Protocol 2: 28.06.2012/  LEC m.m. #7/1  3802: Protocol 1: 21.03.2011/  LEC m.m. #24, Protocol 2: 28.06.2012/  LEC m.m. #7/1  3803: Protocol 1: 21.03.2011/  LEC m.m. #24, Protocol 2: 31.07.2012/  LEC m.m. #53  3804: Prot 1: 10.05.2011/  LEC m.m. #18/11, Prot 2: 27.07.2012/  LEC m.m. #19/12  3805: Prot1: 01.04.2011/  LEC m.m. #24, Prot 2: 22.06.2012/  LEC m.m. #49 |
| 3802 (Local) | EC at Federal State Budgetary Institution |  |
| 3804 (Local) | LEC at Federal State Budgetary Institution |  |
| Serbia |  |  |
| 3901 (Local) | Univerzitetska dečja klinika, Etički odbor | 017-3397/2 |
| 3902 (Local) | Institut za zdravstvenu zaštitu majke i deteta Srbije “Dr Vukan Čupić”, Etički Odbor | 8484/3 F |
| Ukraine |  |  |
| 4102 (Central) | Central EC of Ukraine |  |
| 4102 (Local) | Local Ethics Committee of Government institution «The Scientific-Practical Children’s Cardiac Center» | LEC 4102: # 1010/12-2 of 21.09.2012, meeting minutes # 20 |
| Australia |  |  |
| 5001 (Local) | The Royal Children’s Hospital Human Research Ethics Committee | HREC30131E |
| China |  |  |
| 5102 (Local) | ShangHai Children Medical Center IRB/IEC | SCMCIRB2012034 |
| 5105 (Local) | Ethics Committee of Guangdong General Hospital | YUEMEDICINEEC[2012](18) |
| 5106 | Ethics Committee of Cardiovascular institute & Fuwai Hospital for Clinical Trials | 2012-392 |
| India |  |  |
| 5302 (Central) | Institutional Ethics Committee – CARE Foundation – Hyderabad-1 | --- |
| South Africa |  |  |
| 6001 (Local) | University of the Free State Research Ethics Committee | REC-230408-011/ETOVS172/2010 |
| 6002 (Local) | University of Pretoria Research Ethics Committee | 211/2010 |
| Israel |  |  |
| 7101 (Local) | Helsinki Committee Rabin Medical Center | 20110018 |
| Mexico |  |  |
| 8401 (Local) | Ethics Commitee Instituto Nacional de Cardiologia (INC) Ignacio Chavez | 10-701 |
| USA |  |  |
| 9101 (Local) | IRB - Columbia University Medical Center | IRB-AAAI0311 |
| 9102 (Central) | IRB – Western | e72142 |

EC, Ethics Committees; IRB, Institutional Review Board.
Reproduced under the terms of the CC BY-NC 4.0 license from Berger *et al.* *Pharmacokinetics* 2017;83(8):1734–44.

## **Table S2.** Changes in echocardiographic parameters from baseline in patients naïve to PAH treatment versus non-naïve patients

| **Parameter** | **Time** | **PAH treatment naïve**  **(N = 22)** | | | **PAH treatment non-naïve**  **(N = 42)** | | |
| --- | --- | --- | --- | --- | --- | --- | --- |
|  |  | **n** | **Mean (SD) baseline*** | **Mean change from baseline (95% CI)** | **n** | **Mean (SD) baseline*** | **Mean change from baseline (95% CI)** |
| **RVFAC** | W12 | 13 | 32.832 (13.061) | -5.044  (-8.906, -1.183) | 14 | 35.046 (12.785) | 0.474  (-11.999, 12.947) |
|  | W24 | 9 | 34.911 (12.289) | -1.579  (-12.948, 9.789) | 13 | 36.761 (14.336) | -4.163  (-12.300, 3.973) |
| **IVCC** | W12 | 16 | 47.401 (22.282) | 5.310  (-1.778, 12.399) | 27 | 50.438 (17.364) | 12.854  (6.291, 19.418) |
|  | W24 | 13 | 47.753 (24.441) | 0.176  (-11.924, 12.275) | 20 | 51.689 (18.843) | 12.043  (1.843, 22.243 |
| **LVEID** | W12 | 14 | 1.485 (0.489) | -0.093  (-0.360, 0.174) | 31 | 1.554 (0.439) | -0.050  (-0.200, 0.100) |
|  | W24 | 10 | 1.369 (0.466) | 0.053  (-0.229, 0.336) | 16 | 1.588 (0.438) | -0.114  (-0.363, 0.136) |
| **LVEIS** | W12 | 15 | 1.667 (0.725) | -0.254  (-0.536, 0.028) | 31 | 1.963 (1.033) | -0.173  (-0.460, 0.115) |
|  | W24 | 11 | 1.448 (0.546) | 0.300  (-0.553, 1.153) | 16 | 2.149 (1.082) | -0.390  (-0.853, 0.072) |
| **E/A ratio mitral valve flow** | W12 | 13 | 1.323 (0.397) | 0.083  (-0.118, 0.283) | 21 | 1.411 (0.474) | 0.063  (-0.122, 0.248) |
|  | W24 | 11 | 1.313 (0.399) | 0.382  (-0.249, 1.014) | 14 | 1.545 (0.488) | 0.075  (-0.172, 0.322) |
| **RVSP** | W12 | 18 | 65.323 (27.712) | 0.004  (-8.575, 8.584) | 26 | 72.460 (22.571) | 1.502  (-9.178, 12.183) |
|  | W24 | 13 | 59.951 (28.500) | -0.487  (-11.333, 10.360) | 19 | 73.394 (25.029) | 0.345  (-14.376, 15.066) |
| **TAPSE** | W12 | 17 | 2.550 (1.026) | -0.187  (-0.399, 0.025) | 26 | 2.657 (1.022) | -0.005  (-0.235, 0.226) |
|  | W24 | 10 | 2.812 (1.025) | -0.324  (-0.964, 0.316) | 18 | 2.484 (0.754) | -0.125  (-0.467, 0.216) |

Data are without imputations.

*Mean (SD) baseline values for the number of patients (indicated in the n= cell of each row) who also had data available for that parameter at Week 12 or Week 24 of the core study.

BSA, body surface area; CI, confidence interval; IVCC, Inferior vena cava size collapse; LVEID, diastolic left ventricular eccentricity index; LVEIS, systolic left ventricular eccentricity index; RVFAC, right ventricular fractional area change; RVSP, right ventricular systolic pressure; TAPSE, tricuspid annular plane systolic excursion.

**Figure S1.** Patient flow diagram


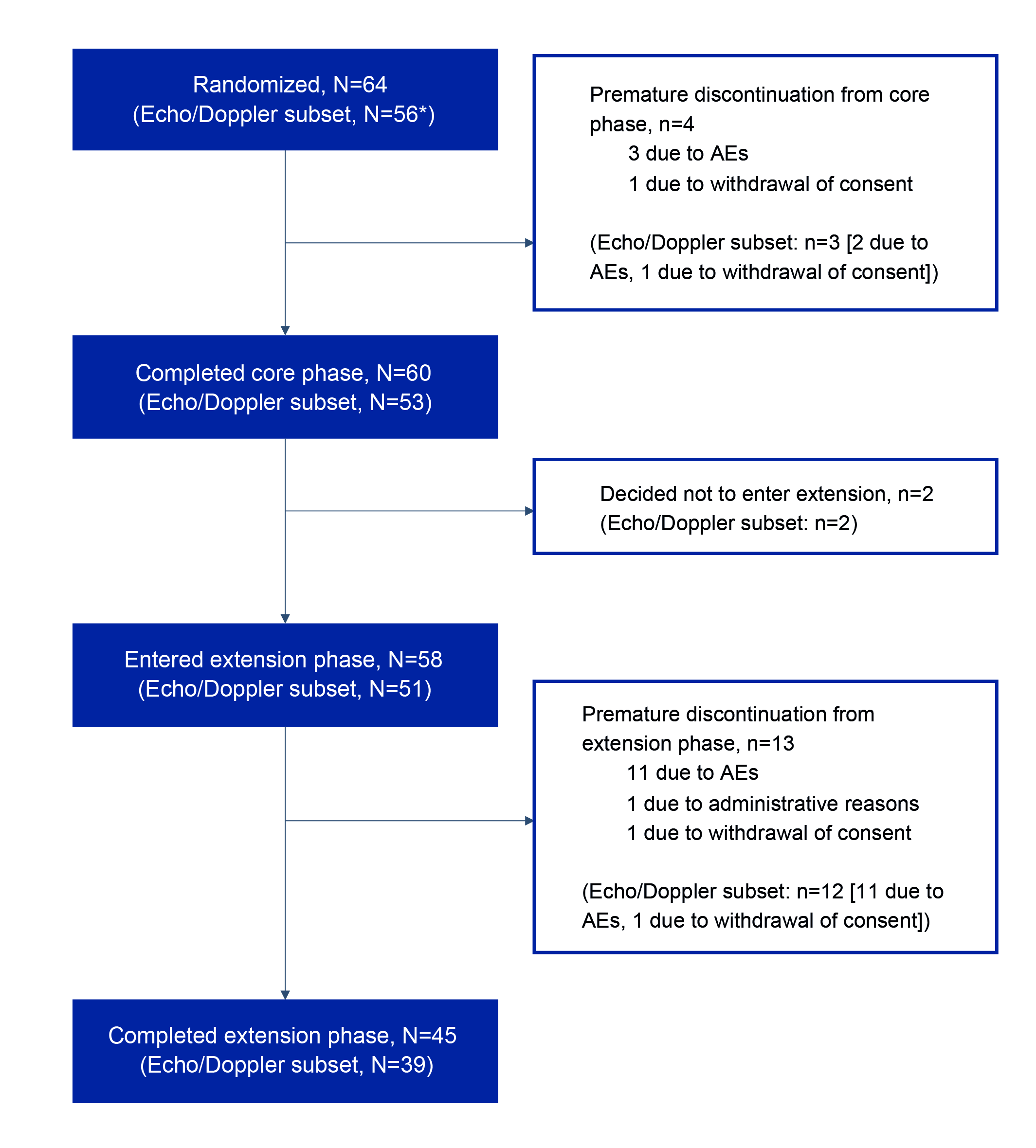


*Eight patients had CHD associated with systemic-to-pulmonary shunts.

There are no patients lost to follow-up in the study (discontinuations due to AEs, consent withdrawal, or administrative reason are not classes as lost to follow-up; patient choosing not to enter extension are not classed as lost to follow-up).

AE, adverse event; CHD, congenital heart disease.

**Figure S2.** Association between worsening from baseline echocardiographic parameters, using thresholds of 10% and 20% for worsening from baseline, and vital status at EOS, at (A) Week 12 and (B) Week 24, respectively (all randomized set; N = 64).

Analysis based on logistic regression. The x-axis shows the estimate of the OR (center tick mark) with its 90% confidence limits (left and right tick marks). The estimate quantifies the increase in odds of experiencing the clinical endpoint in case of a worsening of the echocardiography parameter, where two different threshold changes (10%, 20%) in echocardiographic values were considered. For each threshold, worsening at Week x was used as an explanatory variable and was defined in binary form (i.e. worsening < threshold and worsening ≥ threshold). An OR with a p-value <0.100 indicates a statistically significant correlation between the echocardiographic parameter and vital status at EOS.

CI, confidence interval; EOS, end of study; IVCC, inferior vena cava size collapse; LVEID, diastolic left ventricular eccentricity index; LVEIS, systolic left ventricular eccentricity index; MVR, E/A ratio mitral valve flow; Nb, number; OR, odds ratio; RVFAC, right ventricular fractional area change; RVSP, right ventricular systolic pressure; TAPSE, tricuspid annular plane systolic excursion.


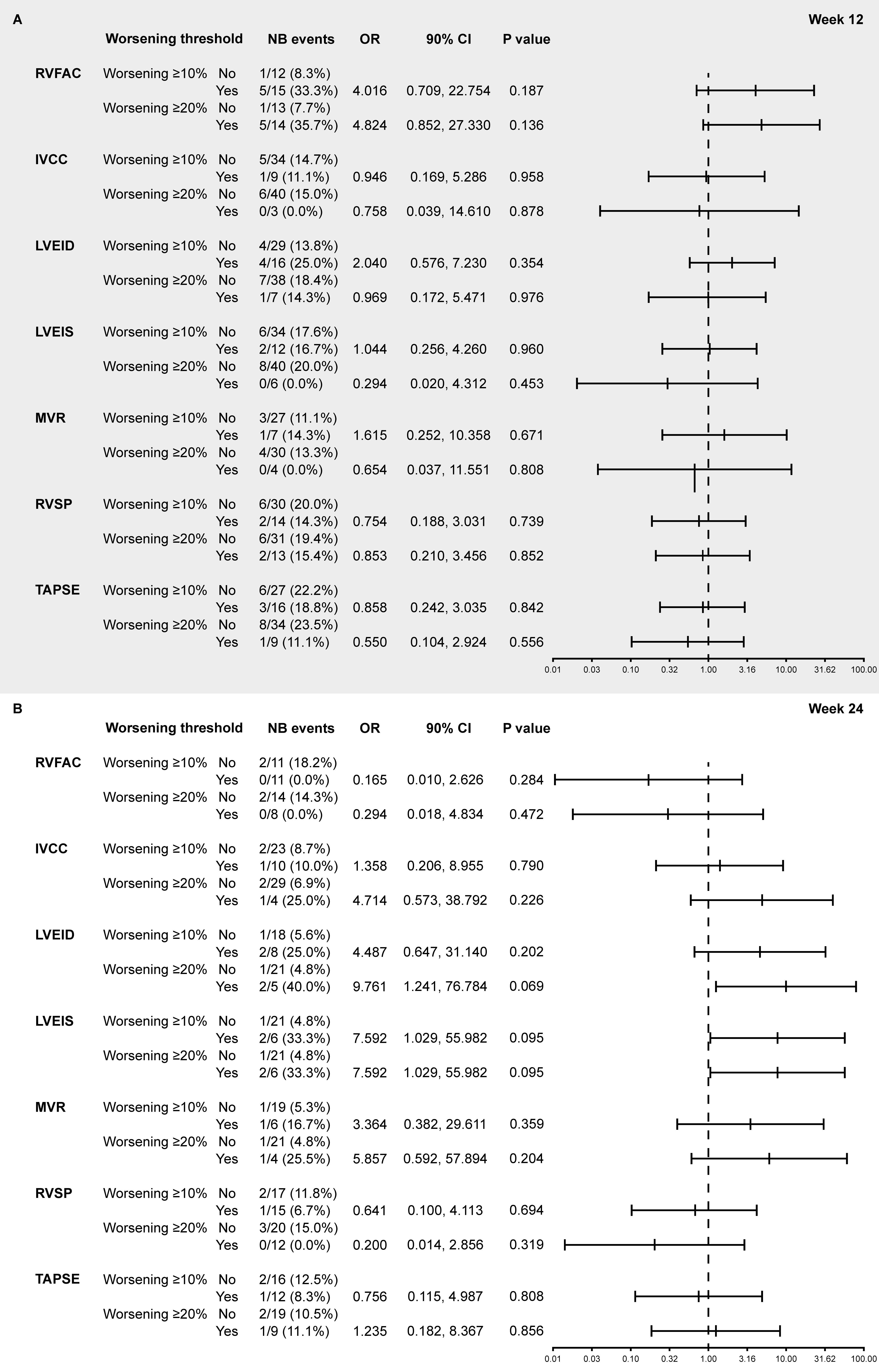


**Figure S3.** Association between worsening from baseline echocardiographic parameters, using thresholds of 10% and 20% for worsening from baseline, and time to death at (A) Week 12 and (B) Week 24, respectively (all randomized set; N = 64).

Analysis based on Cox models. The x-axis shows the estimate of the HR (center tick mark) with its 90% confidence limits (left and right tick marks). The estimate quantifies the increase in risk of experiencing the clinical endpoint in case of a worsening of the echocardiography parameter, where two different threshold changes (10%, 20%) in echocardiographic values were considered. For each threshold, worsening at Week x was used as an explanatory variable and was defined in binary form (i.e. worsening < threshold and worsening ≥ threshold). A HR with a p-value <0.100 indicates a statistically significant correlation between the echocardiographic parameter and the time to death up to EOS.

CI, confidence interval; EOS, end of study; HR, hazard ratio; IVCC, inferior vena cava size collapse; LVEID, diastolic left ventricular eccentricity index; LVEIS, systolic left ventricular eccentricity index; MVR, E/A ratio mitral valve flow; RVFAC, right ventricular fractional area change; RVSP, right ventricular systolic pressure; TAPSE, tricuspid annular plane systolic excursion.


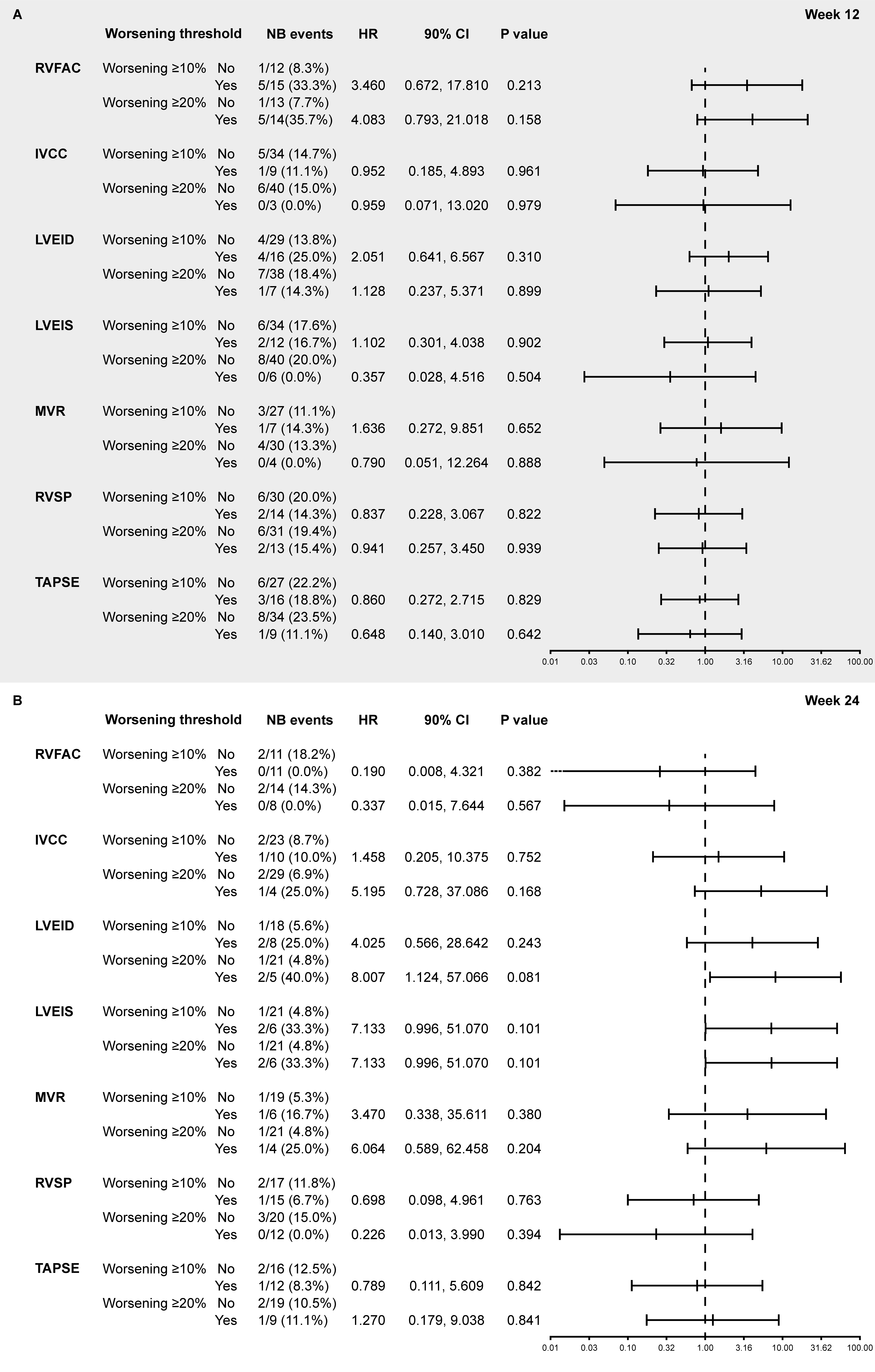


**Figure S4.** Association between worsening from baseline echocardiographic parameters, using thresholds of 10% and 20% for worsening from baseline, and time to PAH worsening at (A) Week 12 and (B) Week 24, respectively (all randomized set; N = 64).

Analysis based on Cox models. The x-axis shows the estimate of the HR (center tick mark) with its 90% confidence limits (left and right tick marks). The estimate quantifies the increase in risk of experiencing the clinical endpoint in case of a worsening of the echocardiography parameter, where two different threshold changes (10%, 20%) in echocardiographic values were considered. For each threshold, worsening at Week x was used as an explanatory variable and was defined in binary form (i.e. worsening < threshold and worsening ≥ threshold). A HR with a p-value <0.100 indicates a statistically significant correlation between the echocardiographic parameter and the time to first PAH worsening up to EOT+7 days.

CI, confidence interval; EOT, end of treatment; HR, hazard ratio; IVCC, inferior vena cava size collapse; LVEID, diastolic left ventricular eccentricity index; LVEIS, systolic left ventricular eccentricity index; MVR, E/A ratio mitral valve flow; RVFAC, right ventricular fractional area change; RVSP, right ventricular systolic pressure; TAPSE, tricuspid annular plane systolic excursion.


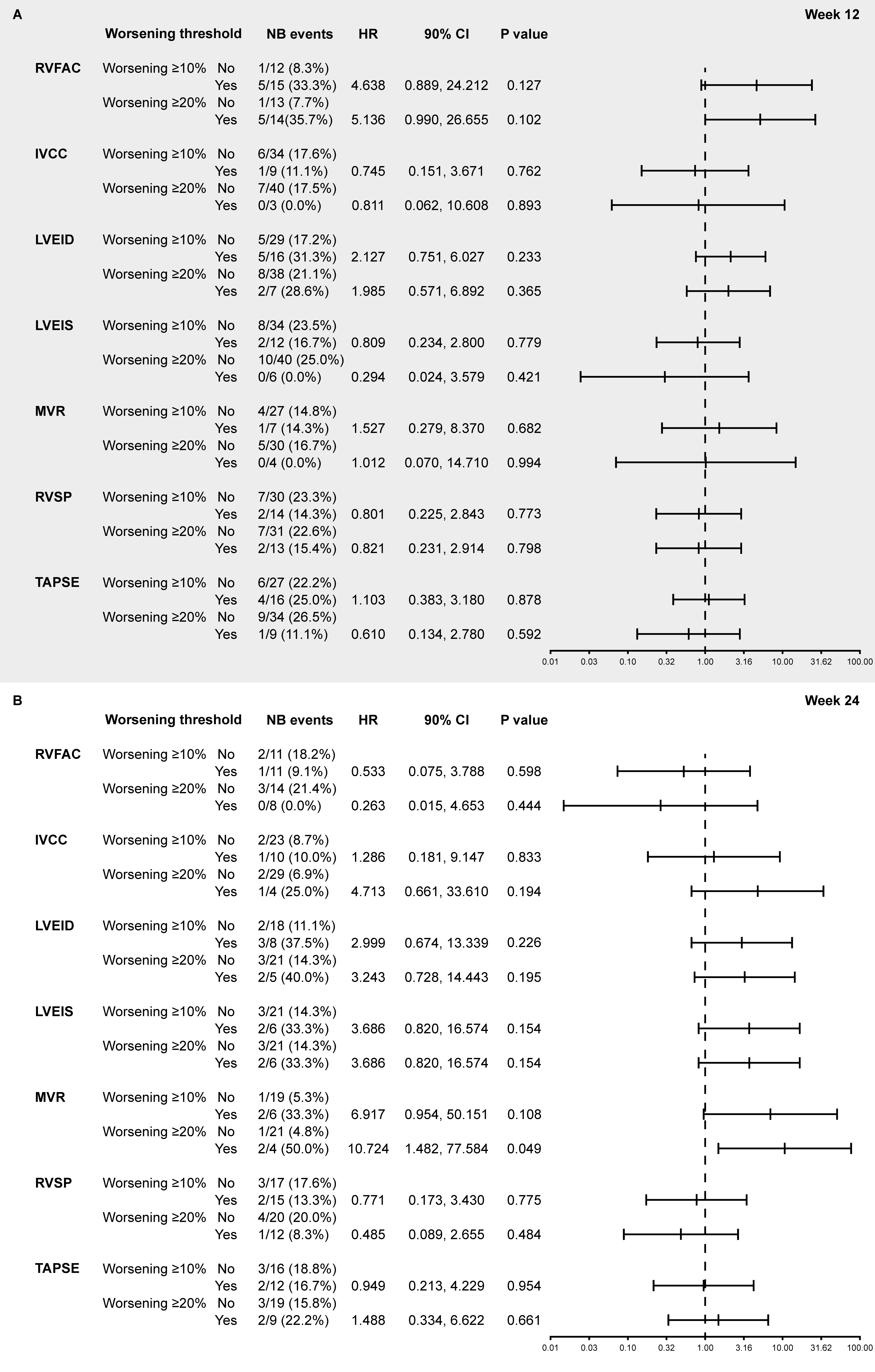

Supplement: Supplementary file 1 [file Table_1.DOCX]
